# Biomarkers of postmenopausal osteoporosis and interventive mechanism of catgut embedding in acupoints

**DOI:** 10.1097/MD.0000000000022178

**Published:** 2020-09-11

**Authors:** Guizhen Chen, Hongyuan Liu, Xiaofeng Wu, Xue Wang, Junquan Liang, Yunxiang Xu

**Affiliations:** a The Bao’an District TCM Hospital, The Affiliated Hospital of Guangzhou University of Chinese Medicine, Shenzhen, China; b Clinical Medical College of Acupuncture, Moxibustion & Rehabilitation, Guangzhou University of Chinese Medicine, Guangzhou, China.

**Keywords:** acupoint thread embedding, bone density, fMRI, postmenopausal osteoporosis

## Abstract

**Introduction:**

Postmenopausal osteoporosis (PMOP), which is a common and frequently occurring age-related metabolic bone disease in perimenopausal women, severely affects patients living quality. Modern medicine therapies for PMOP have several problems such as side reactions, low compliance, and high costs. Thus, nonpharmacological modality is urgently needed. Although acupoint thread embedding treatment is widely used in clinical practice, there is no persuasive evidence of its effect on increasing bone mass for PMOP. This experiment aims to investigate the efficacy and safety of acupoint thread embedding on PMOP and elucidate the correlations among brain neural activation, bone mineral density (BMD), and clinical outcomes with magnetic resonance evidence, thus to explore its neural mechanism.

**Methods:**

This parallel designed, exploratory randomized, controlled, assessor-statistician-blinded, positive medicine clinical trial will include 70 participants with PMOP recruited from 2 traditional Chinese Medicine hospitals. These participants will be randomly allocated to a treatment group (Group Embedding) and a control group (Group Medication) in a 1:1 ratio. Participants in the treatment group will receive acupoint thread embedding treatment once 2 weeks in the following predefined acupoints: Shenshu (BL23), Sanyinjiao (SP6), Guanyuan (RN4), Ganshu (BL18), Dazhu (BL11), Xuanzhong (GB39), Zusanli (ST36), and Pishu (BL20). Meanwhile, the participants in the control group will take 0.3 mg Climen tablet orally, 1 tablet/day; every month has a schedule of the 21-day-continuous-taking-medicine period, and 7-day tablet-free period. There is a study period of 3 months and a follow-up period of 1 month for each group. The primary outcomes will be the following therapeutic indexed: Short-Form of McGill Pain Questionnaire (SF-MPQ), Osteoporosis Symptom Score during the observation period and follow-up period. The secondary outcomes will be Osteoporosis Quality of Life Scale (OQOLS), 16-item Assessment of Health-Related Quality of Life in Osteoporosis. In addition, functional magnetic resonance imaging (fMRI) scans and bone density test will be done before and after the observation period to show cranial neuroimaging changes. All the outcomes will be evaluated before and after treatment. The safety of interventions will be assessed at every visit.

**Discussion:**

We present study design and rationale to explore the effectiveness and neural mechanism of acupoint thread embedding for PMOP through these outcomes.

**Trial registration:**

Chinese Clinical Trial Registry, ChiCTR-INR-17011491.

## 1 Introduction

The definition of postmenopausal osteoporosis (PMOP) is extremely reduced bone density after menopause.^[1]^ As the menopause period occurs younger nowadays, the PMOP also occurs more frequently. It is common in the population over 50 in China with a prevalence of 20.7% and has a high recurrence rate.^[2,3]^ PMOP patients would have several symptoms such as pain in waist and legs, limb weakness, hunchback, and easy fracture. Due to these symptoms, PMOP severely affects patients’ living quality, bringing a heavy financial burden on families and society.^[4]^ Postmenopausal women are the high-risk group of osteoporosis. They are at a special time when disease intervention is particularly important.

Modern medicine therapies and clinical medications are both efficacious in the treatment of PMOP. However, modern medicine therapies toward PMOP such as hormone replacement therapy (HRT), nonhormonal medication therapy have many flaws, such as the variety of side effects, high costs, cancer risk, drug dependence, and so on.^[5]^ Clinical medications toward PMOP such as bisphosphonates, calcitonins also have many flaws, such as renal damage risk, low compliance, the variety of side effects, high costs, and so on. From a clinical point of view, a strong need for effective, few side effects, low cost, and tolerable therapeutic treatments for PMOP still exists. Therefore, investigating the pathogenesis and seeking for valid curative measures are quite essential to postmenopausal patients.

Studies have reported that the etiology of PMOP is generally considered to be the decline in ovarian function and the decrease in estrogen levels in postmenopausal women. These will lead to hypothalamic-pituitary-ovarian axis dysfunction and affect bone metabolism. Some randomized controlled trial (RCT) studies have shown that acupoint thread embedding therapy (also called acupoint catgut embedding therapy and the name of the therapy changed due to the development of suture) can reduce the pain level of PMOP patients. It can also regulate hormone secretion levels, E2 level, reproductive endocrine, and bone metabolism of postmenopausal women.^[6–9]^ Until now, the exact mechanism of PMOP is still not clear.^[10]^

For traditional Chinese medicine (TCM), acupoint thread embedding treatment is widely used to treat a variety of health problems, including ostealgia from PMOP.^[11]^ However, there is no persuasive evidence of its effect on PMOP. Hence, some sensible evidence of its effectiveness and safety in treatment of PMOP is urgently needed. Imaging techniques can provide an effective approach to study the functional brain region. With the help of functional magnetic resonance imaging (fMRI), the changes in neural activity in different brain regions before and after the study can be gathered. Thus, the pathogenesis of PMOP may be revealed as well as the neural mechanism of acupoint thread embedding treatment.

## 2 Methods/design

### 2.1 Objectives

This study aims to investigate the efficacy and safety of acupoint thread embedding treatment on PMOP and explore its neural mechanism.

### 2.2 Trial design and study setting

**Figure 1:**
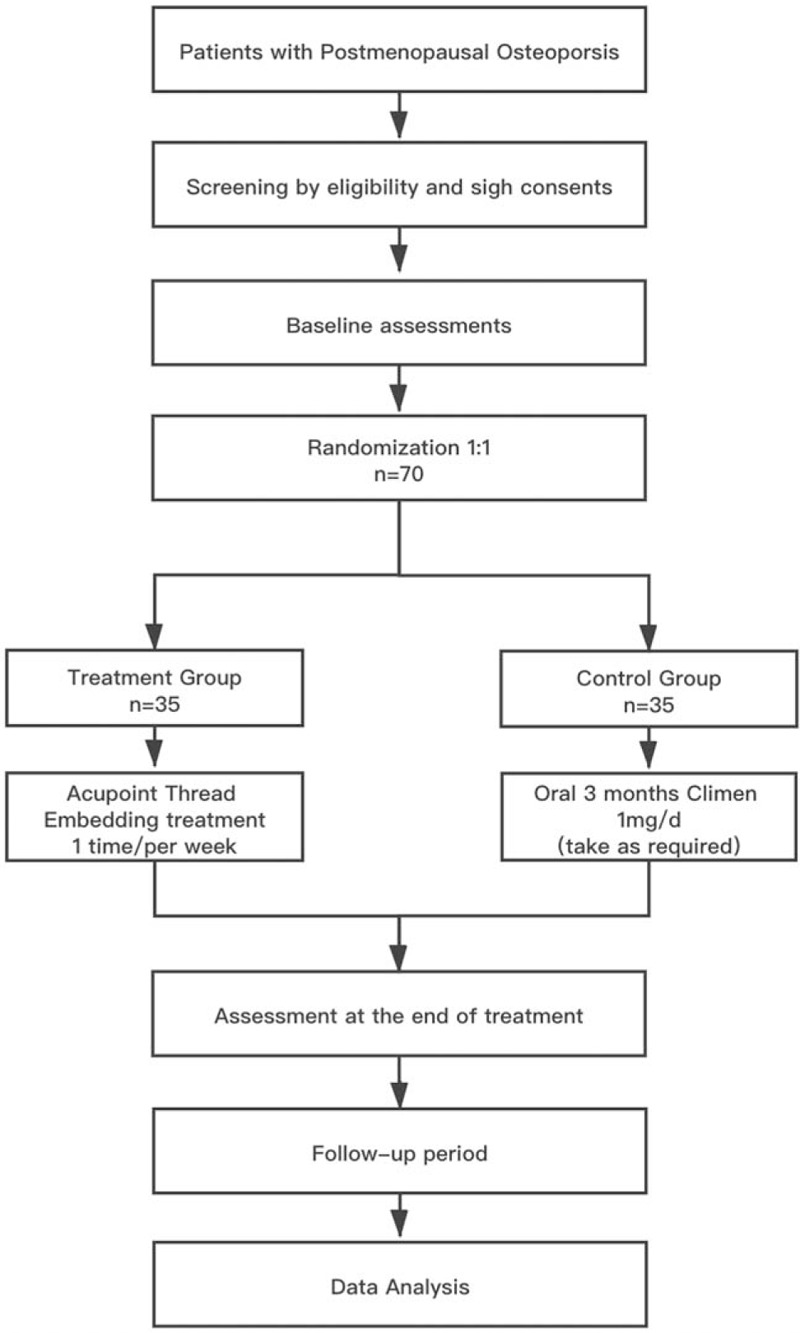
Trial design and study flowchart.

This study is a parallel designed, exploratory randomized, controlled, assessor-statistician-blinded, positive medicine clinical trial. Seventy participants with PMOP will be randomly assigned to the treatment group (taking acupoint thread embedding treatment, n = 35) and a control group (taking Climen treatment, n = 35) in a 1 : 1 ratio. The trial design and study flowchart are shown in Fig. 1.

### 2.3 Recruitment

Two Chinese Medicine hospitals located in Guangdong, China, namely the Bao’an District TCM Hospital, the Affiliated Hospital of Guangzhou University of Chinese Medicine, and Guangdong Provincial Hospital of TCM, will recruit participants in the clinical practice setting. Clinical trial information will be posted on the bulletin board of each hospital and nearby communities, in WeChat subscription, in social software.

### 2.4 Eligibility criteria: inclusion criteria

(1) Female subjects aged 45 to 70 years and have natural menopause for more than 2 years;
(2) After the dual-energy X-ray absorptiometry, *T* value -2.5 ∼ -5.0 (L2-L4), and meet the diagnostic criteria of TCM syndrome;
(3) Subjects voluntarily participated in the study and signed informed consent;
(4) Subjects who can be able to cooperate and complete the fMRI scan.

### 2.5 Eligibility criteria: exclusion criteria

(1) Patients whose age is under 45 or over 70 years, natural menopause is fewer than 2 years;
(2) Patients who have a history of hypersensitive reactions to acupoint thread embedding treatment or Climen;
(3) Combined with other diseases affecting bone metabolism: endocrine diseases (gonads, adrenal glands, parathyroid glands, and thyroid diseases, etc), rheumatoid arthritis and other immune diseases, affecting the absorption and regulation of calcium and vitamin D, digestive tract and kidney diseases, malignant diseases such as multiple myeloma, long-term use of glucocorticoids or other drugs that affect bone metabolism, as well as a variety of congenital and acquired bone metabolism disorders;
(4) Combined with malignant tumors, cardiovascular, cerebrovascular, liver, kidney, hematopoietic system, severe primary diseases, severe skin diseases, mental disorder patients;
(5) Patients who have received osteoporosis medication (excluding calcium supplementation) within 6 months or have long-term use of drugs that may affect bone calcium metabolism;
(6) Patients who have pathological changes in the brain, such as brain tumors;
(7) Patients who have MR examination contraindications: such as aneurysm clips, implanted nerve stimulators, pacemakers, automatic defibrillators, cochlear implants (electrodes), or visual foreign bodies;
(8) Patients who have a history of abuse of substances such as alcohol and drugs, or a history of dependence on alcohol or drugs;
(9) Patients who are late disabled, deformed;
(10) Ineligibility for the trial judged by the clinical trial investigator.

### 2.6 Subject withdrawal criteria

(1) Violation of the inclusion criteria or fulfillment of the exclusion criteria;
(2) Serious adverse events occurred during the treatment;
(3) Withdrawal of consent by the subject or a legal representative;
(4) Those who were unable to adhere to these treatments due to other medical conditions during the study period;
(5) Violation of the clinical trial protocol by the investigator or the subject;
(6) The loss to follow-up;
(7) Use of medications or treatments that can affect the results of the clinical trial without permission from the investigator;
(8) Inappropriate progress as judged by the investigator.

### 2.7 Randomization and allocation concealment

The randomization number will be generated by PEMS 3.1 and made into random cards. The randomization number will be sealed in a predetermined randomization opaque envelope. The patients’ screening sequence numbers will be printed on the outside of the envelope, whereas the group names will be printed inside. The envelope containing an allocation sequence number for each patient will be opened right after each patient is verified to meet the eligibility criteria and has signed the written informed consent form. If any error or disclosure regarding randomization occurs, a new randomization sequence will be generated from the problematic serial number and applied to subsequent patients.

### 2.8 Blinding

Considering the significant difference of treatment and the specificity of acupoint thread embedding treatment, double-blinding of the practitioners and participants is unfeasible. The assessor will be blinded. The treating physician will not assess the outcome measures. The assessor will be instructed to refrain from talking about the treatment in order to maintain blinding. The statistician and data collector will also be blinded. To guarantee the integrity of this trial, the study will strictly conduct randomization allocation and appoint different people to perform therapy, assessment, and statistical analysis according to the principle of blinding.

### 2.9 Intervention

#### 2.9.1 Study schedule

The study schedule is presented in Table 1. At the screening visit, participants will sign the informed consent form. In a baseline questionnaire, the following information will be obtained: age, occupation (physical/nonphysical), education level, last menstrual period, previous use of acupuncture, and expectation to acupoint thread embedding treatment. Moreover, Short-Form of McGill Pain Questionnaire (SF-MPQ), Osteoporosis Symptom Score, Osteoporosis Quality of Life Scale (OQOLS), 16-item Assessment of Health-Related Quality of Life in Osteoporosis (ECOS-16) must be completed at the baseline (week 0). Thereafter, study subjects will be randomized. There is a 3-month study period and a 1-month follow-up period for each group. In the study period, SF-MPQ, OQOLS, ECOS-16, and Osteoporosis Symptom Score will be evaluated every 4 weeks. fMRI and bone density test will be tested 2 times: in week 0 and week 12.

**Table 1:**
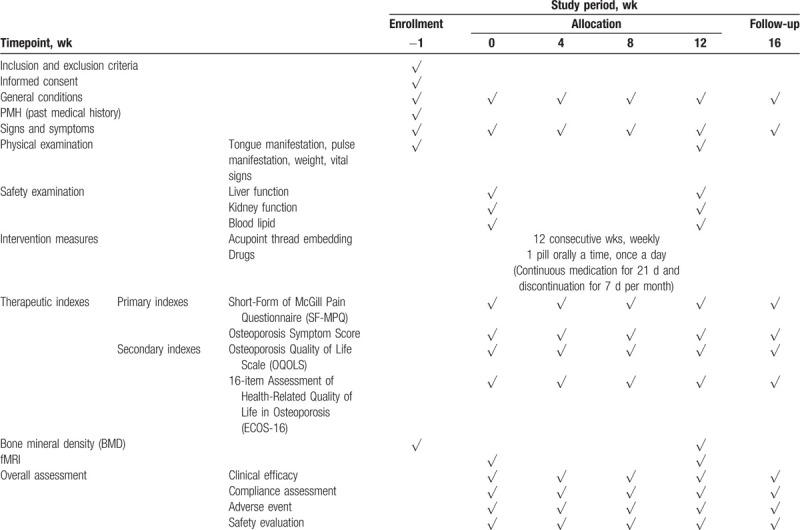
Trial flow and time schedule: enrollment, interventions and assessments.

#### 2.9.2 Treatment group intervention

Acupoint thread embedding treatment will be applied to patients in the treatment group fortnightly, a total of 6 times during 3 months. Main acupoints are BL23, SP6, and RN4. If it is a liver-kidney deficiency case, BL18, GB39, BL11 will be added. If it is a kidney yang deficiency case, BL20, ST36 will be added. In each treatment, RN4 is a required acupoint. The rest acupoints can be used in the alternation of the left/right side. The location method is according to *Location of Acupoints* the National Standards of P.R promulgated by the State Bureau of Technology Supervision. The operating method follows the National Standards of P.R (GB/T 21709.10-2008) manipulations of acupuncture and moxibustion—Part 10 Acupoint catgut embedding. Details about the intervention group interventions and the acupoints location are described in the Standards for Reporting Interventions in Clinical Trials of Acupuncture (STRICTE) checklist in Tables 2 and 3.^[12]^

**Table 2:**
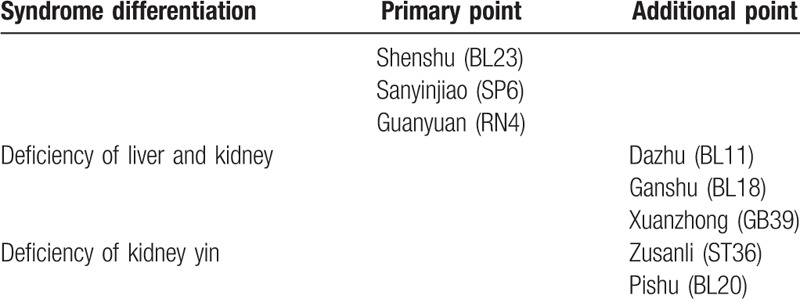
Acupoints selects.

**Table 3:**
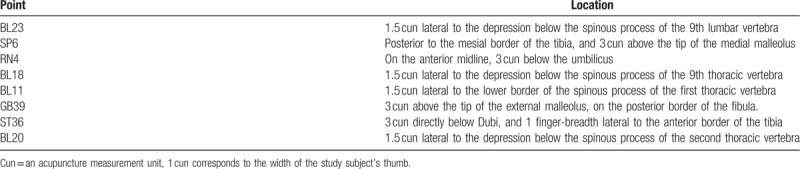
Acupoints location.

Medical material information includes that the needles will be sterile disposable No.8 injection needles, size 0.8 × 38TW LB (Zhejiang Kangkang Medical Devices Co., Ltd., Chumen Town, Yuhuan County). The core needles will be sterile disposable No.28 acupuncture needles, size 0.35 × 50 mm (Rui Qier), used after their pinpoints rubdown. The thread will be absorbable surgical suture (medical thread), size3/0 (Shanghai Pudong Jinhuan Medical Products Co., Ltd., Shanghai, China).

Operating method (implantation with needles): According to National Standards of P.R (GB/T 21709.10-2008) manipulations of acupuncture and moxibustion—Part 10.

(1) Cut the 3/0 thread into 1.5 cm segments and soak them in disinfectant.
(2) During the treatment, patients take prone position to get back-shu point implantation and supine position to get RN4, SP6, GB39, and ST36 implantation. On the basis of location method above, disinfect routinely at acupoint and its skin around with Anerdian, take one self-made embedding needle, stick the core into needle tubing, and then pull out the core about 2 cm, implant one 1.5 cm sterile thread into the front of the tubing, and tighten or pinch the local skin around the acupoint with left thumb and forefinger, stick the needle into the acupoint rapidly with the right hand, and the lift and down the needle until receiving qi. Push the core and withdraw the tubing slowly, and finish implanting the thread deep down the acupoint. Hold pressure for a moment after withdraw the needle, check for exposure of thread out of the skin and bleeding, cover the wound with a piece of gauze or band-aid for 1-2 days.
(3) Direction, angle, and depth of implantation: BL23, SP6, RN4, GB39 are required vertical puncture into the acupoint for about 0.8 to 1.0 cun (approximately 20--25 mm); ST36 is required to vertical puncture into the acupoint for about 1.0 to 1.5 cun (approximately 25–40 mm); BL20 is required vertical puncture into the acupoint for about 0.5 to 0.8 cun (approximately 13–20 mm); BL18, BL11, DU4 is required oblique puncture toward the spine into the acupoint for about 0.5 to 0.8 cun (approximately 13–20 mm).

#### 2.9.3 Control group

The participants in the control group will be prescribed oral administration of Climen (2 mg Estradiol 17β-valerate and 1 mg Cyproterone 17α-acetate) tablets (1 tablet 30 minutes before sleep once per day for 21days and then stop medicine for a week; lasting for 12 weeks). The Climen tablets are produced by Schering GmbH Und Co. ProduktinosKG (Guangzhou, China) (Approval No. J20130006).

#### 2.9.4 Adverse events

During the research, some adverse reactions may occur. Researchers will monitor every patient with adverse reaction.

The following adverse reactions may happen after the acupoint thread embedding:

(1) Local reaction: mostly some sterile inflammation with inflamed hot pain within 5 days. Some cases may be more severe, like a small amount of milky-white exudation at the embedding spot caused by fat liquefaction because of thread stimulation. Circumstances above should not require any special treatment.
(2) General reaction: some patients may experience temperature rising after the treatment 4 to 24 hours, which mostly around 38°C. It will fade away in 2 to 4 days by itself. Usually, hemogram of each patient may rise in different levels, and it will return to normal in 3 to 5 days.

If adverse reactions occur, the following treatment will be used.

(1) Pain at implantation acupoint: Apply hot compress.
(2) Local swelling, progressive pain, and fever 3 to 4 days after the implantation: Lack of strict asepsis and inappropriate protection of the wound could cause secondary infection, which mostly occurs some inflammatory symptoms. Apply hot compress and anti-infective treatment would help.
(3) Nerve injury: It is usually caused by incorrect operation or excessive stimulation or carelessness, and can be avoided with the careful operation.
(4) Hemorrhage: It is usually caused by the puncture on the vessels or excessive stimulation, in which case pressure dressing on the puncture point would stop the bleeding.
(5) Thread allergy: local itching or swelling or fever can be remitted by antianaphylaxis treatment.

Climen, which includes estradiol and cyproterone acetate, is one of the estrogen and progestogen/anti-androgen (HRT). It is used in HRT for women with an intact uterus, used to substitute for a lack of estrogen production by the ovaries during menopause. It helps prevent postmenopausal bone loss (osteoporosis) and reduce risk of fracture. The most commonly reported side effects when taking Climen include breast tenderness, breast pain, abdominal pain, nausea, edema, headache, depression, changes in body weight, and changes in libido.

#### 2.9.5 Concomitant treatment

Other interventions such as medications, injections, physical therapy, surgical procedures, acupuncture, cupping, and moxibustion will not be permitted during the 12-week treatment period (not applicable to the follow-up phase). However, any concomitant medications are taken by the subject within 4 weeks before trial participation, which will not affect the interpretation of the trial outcomes, will be allowed at the discretion of the investigator.

#### 2.9.6 fMRI examination procedure

The fMRI examination (PRISMA 3.0T Magnetic Resonance Imaging Scan; Siemens Shanghai Medical Equipment Ltd.) will be performed to collect brain images offering more visualized proof. During the scanning procedure, subjects will be asked to keep quiet and their eyes closed. In the meantime, they need to keep their mind empty but avoid sleeping. The researcher will check whether the participants are awake during the process. The MRI protocol for this study consists of the following parameters: T1 scanning: repetition time (TR) = 2300 ms, echo time (TE) = 2.32 ms, flip angle = 8°, the field of view (FOV) = 240 × 240 mm^2^, matrix size = 256 × 256 mm^2^, voxel size = 0.9 × 0.9 × 0.9 mm^3^, 192 sections, 0.9 mm thickness, 0.45 mm slice gap, 200 Hz/pixel bandwidth. MRS scanning: TR = 1700 ms, TE = 135 ms, 1 mm thickness, no slice gap. DTI scanning: matrix size = 128 mm × 128 mm, FOV = 220 mm × 220 mm, 3.8 mm slice thickness, 33 slices centered on the corpus callosum, b-value = 1000 s/mm^2^. Blood oxygen level dependent scanning: TR = 3000 ms, TE = 30.0 ms, flip angle = 90°, the FOV = 192 × 192 mm^2^, voxel size = 3 × 3 × 3 mm^3^, 36 sections, 3.0 mm thickness.

#### 2.9.7 Bone density test

The bone density test (DPX Bravo Dual-energy X-rays Absorptiometry; General Electric Co., Ltd.) will be performed to collect bone mineral density (BMD) data.

### 2.10 Outcome measure: primary outcomes

Outcomes will be evaluated using multiple indexes. The primary outcomes are measured by the following therapeutic indexes: Short-Form of McGill Pain Questionnaire (SF-MPQ), Osteoporosis Symptom Score during the observation period (3 months) and follow-up period (1 month). After baseline time, they will be tested at week 0, week 4, week 8 and week 12, and during the follow-up period.

### 2.11 Outcome measure: secondary outcomes

Outcomes will be evaluated using multiple indexes. The secondary outcomes are measured by the following therapeutic indexes: Osteoporosis Quality of Life Scale (OQOLS), 16-item Assessment of Health-Related Quality of Life in Osteoporosis (ECOS-16). All the outcomes will be evaluated before and after treatment. The safety of interventions will be assessed at every visit. After baseline time, they will be tested at week 0, week 4, week 8 and week 12, and during the follow-up period.

### 2.12 fMRI and BMD

fMRI will focus on the activity of the hypothalamus, limbic system, and the connection among neuron circuits to reflect the changes of the brain's function of PMOP patients before and after treatment. BMD will test the density to certain the severity of osteoporosis. fMRI scans and bone density test will be done before and after the observation period to show cranial neuroimaging changes.

### 2.13 Safety assessment

At every visit, the physical examination will be performed, and adverse events will be checked. Blood samples will be collected at week 0 and week 12. Blood routine test, liver function (alanine aminotransferase), and kidney function (serum creatinine) will be done at the beginning and the end of the intervention period.

Possible adverse events due to acupoint thread embedding include allergy, local infections, subcutaneous hematoma, and so forth. Any adverse events or reactions that occur during the study process will be addressed properly and analyzed by the investigator. Serious adverse events associated with the trial should be reported to the principal investigator immediately. All unexpected and unintended responses will be documented as adverse events by the researcher at every visit, even if they are not necessarily related to the acupuncture intervention. The details of adverse events will be carefully recorded in the CRF by the corresponding research staff.

### 2.14 Statistical analysis

Image data processing includes using methods such as independent component analysis (ICA) and function binding, applying professional software such as MRIcro for windows, Xjview 8.1, SPM 12, and Matlab 5.1.

Statistical analysis technique is used to apply a statistical test to the difference of prior-treatment and posttreatment, and to examine the curative effect difference within groups and among groups of all curative effect indexes. We will adopt the 2-sided test for all statistical tests. It is considered that the tested differences are statistically significant if *P* value is less than or equal to .05. If *P* value is greater than or equal to .05, the tested differences are not statistically significant. Details are as following.

Measurement data includes the *t* test will be adopted to run comparison among groups. When it does not comply with the normal distribution, we will choose Wilcoxon rank-sum test. Similarly, we will run paired *t* test toward the difference of prior-treatment and post-treatment, and it will be changed into Wilcoxon rank-sum test, while it does not comply with the normal distribution.

Enumeration data: We will use the Chi-square test, calibration Chi-square test, and Fisher exact method to run comparison among groups.

Ranked data: Wilcoxon rank-sum test will be applied to run comparison among groups and signed rank-sum test to run comparison within groups.

### 2.15 Quality control

To ensure the control group's compliance, participants will receive the Climen tablets of a week dose at the face-to-face interview every week, and sign on the medicine record of the CRF. In addition, researchers will communicate with them every day to visit their medication via call, WeChat, short message, etc.

All of the acupuncturists who take part in this trial have gained medical licenses in China and have been well trained according to the trial's procedure under the guidance of senior acupuncturists. All of the investigators were qualified to perform this study after they participated in special training classes about this trial and were tested to well understand the research. For instance, they were trained to use the randomization number table, to fill in the case report form, to identify the correct acupoints, to manipulate the needles, and so on. In addition, to ensure the quality of the study, the clinical monitors will check the process of the trial and document the details of the processes once a week. Moreover, monitors nominated by the principal investigator will check the accuracy and validity of the original data from the clinical center. Regular meetings will be held to consider the recruitment rate, adverse events, protocol violation, difficulties and problems emerging during the study, and so forth.

### 2.16 Ethics approval and registration

This clinical trial will be carried out in accordance with the Declaration of Helsinki, as well as be reviewed and approved by the Medical Ethics Committee Board of Affiliated Bao’an TCM Hospital of Guangzhou University of Chinese Medicine (Grant No: 20160923). All of the participants will be requested to sign the written informed consent form before randomization. The present study is financially supported by the National Natural Science Foundation of China (Grant No: 81574064; 81473755), the Shenzhen Science and Technology Planning Project (grant No: JCYJ20170306152650625), and the Shenzhen Bao’an Traditional Chinese Medicine Hospital Research Program (Program No. BAZYY20200609). The clinical trial was registered in the Chinese Clinical Trials Registry in May 2017.

## 3 Discussion

This study is a randomized, controlled, assessor-statistician-blinded, positive medicine clinical trial, funded by the National Natural Science Foundation of China. The purpose of this project is to verify the efficacy and safety of acupoint thread embedding on PMOP. The goal is hoped can be achieved by evaluating BMD measurement, fMRI, clinical integral scale, etc. This study is based on syndrome differentiation of viscera (kidney syndrome differentiation) to determine the acupoints used in thread embedding therapy. The researchers searched papers for nearly 10 years and found that the treatment of osteoporosis based on viscera syndrome differentiation (kidney syndrome differentiation) is effective. It has been investigated that many cases of osteoporosis patients in Nanjing responded to the treatment of “ tonifying kidney and benefiting marrow” method. In the treatment of osteoporosis, the method has achieved good evaluation.^[13]^ In TCM theory, “kidney controls bones and marrow moistens bones,” which means that the kidney essence can improve the growth and development of human, and bone formation. Broadly speaking, the kidney essence consists of marrow, spinal cord, and brain. And the brain is the “marrow sea”; it plays a leading role in the growth and development of bone. The kidney affects the growth and development of bone by its close connection with the brain. Combining with the theory of “brain-bone mass regulation” in modern medicine,^[14]^ the researchers believe that the method of “tonifying kidney and benefiting marrow” affects kidney and brain, then influences bone metabolism, and is a symptomatic treatment for osteoporosis.

Furthermore, the previous study found that acupoint stimulation has certain effect on perimenopausal syndrome.^[9]^ Therefore, the study is designed by thread embedding therapy at acupoints after syndrome differentiation, in order to provide evidence for the clinical efficacy and the effect of the brain-bone mass regulation mechanism in the nervous center.

In addition to using SF-MPQ, OQOLS, ECOS-16, and osteoporosis symptom scores to evaluate clinical efficacy, the study also chose BMD measurement and MRI scan to provide clear, effective, and visible evidence to verify the efficacy and safety of acupoint thread embedding on PMOP.

The study found several evidences about acupuncture (acupoint stimulation) treatment of PMOP^[9,15]^ and changes in bone mass caused by changes in brain endocrine hormones. Studies have shown that when the original bone resorption and bone formation balance is broken, after that, bone resorption increases, bone formation decreases, and osteoporosis is induced or aggravated.^[16,17]^ Bone physiology is coordinated by brain control and is associated with the transmission of leptin signaling.^[18]^ Hypothalamus release neurotransmitters via neuropeptides and monoamines, which regulate the secretion of gonadotropin-releasing hormone, growth hormone-releasing hormone, and thyrotropin-releasing hormone, interfering with bone growth and metabolism.^[19]^ Decreased expression of protein kinase C, bone morphogenetic protein, and its signal transduction molecule in osteoporosis model rats can significantly increase BMD.^[20,21]^ Acupoint embedding may regulate the neuroendocrine function by regulating the secretion of gonadotropin-releasing hormone (GnRH), growth hormone-releasing hormone (GHRH), and thyroid-stimulating hormone-releasing hormone (TRH) in the arcuate nucleus of the hypothalamus through monoamine neurotransmitters, then adjust the bone mass.^[19]^ Acupuncture (acupoint stimulation) can improve the symptoms of PMOP to some extent, and found that the brain's hormone levels have changed. Therefore, the researchers hypothesized that acupoint thread embedding therapy can improve the symptoms of PMOP and designed the study.

Nevertheless, there are also limitations in this study. Concerning the unfeasible blinding to participants, one limitation is the possibility of a high risk of bias regarding blinding by unconcealed allocation. Therefore, we strictly conduct randomization and separate the acupuncturist, assessor, and statistician. Meanwhile, patients divided into the acupuncture intervention group may have more opportunities to make contact with their acupuncturists and establish a good doctor--patient relationship rather than those of the medicine control group, which is likely to improve the therapeutic effect. Hence, we will maintain close contact with patients in the control group by telephone, Internet, and so on to minimize this potential bias. At the same time, BMD is one of the important indicators of this study, but the change of BMD is not visible in the short term, which does not match the 3-month cycle of this study. However, as the main diagnostic index of PMOP, BMD cannot be ignored, so it remains in the experiment. To this end, researchers seek breakthroughs in neuroimaging, with a view to finding early imaging markers through fMRI before and after treatment, and judging the efficacy with other indicators such as symptom scale.

In conclusion, combining fMRI, BMD, and subjective symptom scale, the relevant outcomes are predicted to verify the efficacy and safety of acupoint thread embedding therapy. We believe that the results will exert a positive effect on acupoint thread embedding treatment for PMOP.

### 3.1 Trial status

The trial is currently in the recruitment phase. Recruitment began on September 12, 2018. We expect the recruitment phase to be complete by the end of 2021. Written informed consent will be obtained from all study patients before study enrollment. After each qualified patient consents to participate in, enrollment and data collection will be started. And informed consent was formulated.

## Acknowledgments

The authors thank the National Natural Science Foundation of China (grant No: 81574064; 81473755), the Shenzhen Science and Technology Planning Project (grant No: JCYJ201703063000328), and the Shenzhen Bao’an Traditional Chinese Medicine Hospital Research Program (Program No. BAZYY20200609).

## Author contributions

GZC conceptualized the study and drafted the manuscript. YXX contributed to the development and refinement of the study protocol. HYL and XFW drafted and revised the manuscript. JQL and WJW made contributions to the neuroimage methods and statistical analysis. All authors reviewed and approved the final manuscript.
